# A Highland Laird's Family
*"The Household of McNeil." By Amelia E. Barr, author of " Jan vedder's Wife," &c. London: James Clarke and Co., 13 and 14, Fleet Street.


**Published:** 1889-03-23

**Authors:** 


### A Highland Laird's Family.*

We confess to a sense of being aggrieved by Mrs. Amelia Barr's last book, "The Household of McNeil." The McNeil is not quite the laird of our dreams. He is as fiery, as proud, as impetuous as any Celt need be; but he has other qualities, doubtless praiseworthy and possibly Celtic, that jar a little on our preconceptions. We cut our romance teeth on Sir Walter Scott, and not even a subsequent perusal of the memoirs of that very mercenary personage, Lusson, Lord Lovat, and the confessions of his cousin, Captain Fraser, could do away with the glamour with which the Wizard had invested the Highlands and the Highlanders. Perhaps it is a weak craving for the picturesque rather than the true that makes us resent the M'Neil adding largely to his income by becoming the owner of a hotel! This practical tinge is hereditary, and we find his daughter Grizelda objecting to a secret marriage chiefly because by so doing she will forfeit the large dowry her father would otherwise give her. Love-sick maids—even maids who out of selfishness and waywardness only dream they are love-sick—do not hesitate at throwing away a trifle of sixty thousand pounds.

Having thus had out our complaint against Mrs. Barr, we may say that "The Household of M'Neil" is a pleasant and interesting story, with clearly-marked characters and a well-constructed plot. Walter, Lord Maxwell—who marries Grizelda M'Neil merely to spite her father, ill-treats her, and finally plots to have her murdered—is as despicable a villain as one need wish to see safely confined within the boards of a book. There is a grim humour in the account of the manner in which he was nursed by Peppo, the bravo whom he had hired to murder his wife, when he was suffering from rheumatic fever. We commend it to our readers as an admirable example of "how not to do it." Maxwell's subsequent conduct, his marriage with another woman, although he is by no means sure that Grizelda is really dead, having a shrewd suspicion that his accomplice Peppo had fooled him; his heartless insolence to the father of his lost wife; his selfish cowardice when his crime is discovered, and Grizelda re-appears—all seems the thoroughly natural outcome of his depraved nature. Horrible as is the narrative of his death from hydrophobia on the lonely island to which he had fled, one feels that his fate was as good as he deserved.

The most winning female character in the story is not Grizelda, the heroine, but her sister Helen, one of those rare, exquisite women who carry about on earth the atmosphere of heaven, but are so much more akin to the higher sphere that we scarcely feel grieved when death claims them from the arms of love. Colin, her lover, is too placid; we hear of his energy and goodness, but we do not realise them. We much prefer Dr. Bradick, the minister, whose talks with McNeil, however didactic in subject, never lose the flavour of humanity. The struggle in McNeil's mind between his love of money and his desire to obey his dead daughter's wish, and the influence of the minister's own spiritual development upon the wavering laird, are well brought out. We must note however, that it is strange a writer so familiar with Scotch habits should make Maxwell ask his sweetheart to meet him in St. Andrew's Kirk with a view to being married privately. A marriage in church is in Scotland, especially among the Presbyterians, the most public possible, and at the date of the story told in "The Household of McNeil," was all but unknown. Was Mrs. Barr anxious to avoid wounding those susceptibilities of Mr. Oscar Wilde which make him object to "a story without an anachronism to speak of?"

* "The Household of McNeil." By Amelia E. Barr, author of "Jan Vedder's Wife," &c. London: James Clarke and Co., 13 and 14, Fleet Street.

### SURFACE ANATOMY.

"A Handbook of Surface Anatomy and Landmarks." By Bertram C. A. Windle, M.A., M.D., Professor of Anatomy in the Queen's College, Birmingham.—The author states that this small book is intended for anatomical students in their first and second years, as a guide to that important portion of the knowledge of the human body which can be gained without the use of the scalpel and forceps. The importance to the student of obtaining a thorough knowledge of medical and surgical landmarks, is at once apparent, and Dr. Windle's book meets a want. It does not aim at portraying surgical anatomy, excepting as regards the surface of the body, and the different orifices. As an introduction to works an anatomy, the value of this little book cannot be overestimated. We are not, however, clear that it should be regarded as a guide to a second year's student. In our view a second-year student should have mastered at least all the elementary portion of anatomy. We can, however, join with the author in remarking that junior students will find the book useful, after having first dissected a part of the body, to study the relations of the various structures on a fresh part, or on the living subject.

